# Synergistic Antibacterial Potential of 6-Pentyl-α-pyrone Lactone and Zinc Oxide Nanoparticles against Multidrug-Resistant *Enterobacterales* Isolated from Urinary Tract Infections in Humans

**DOI:** 10.3390/antibiotics11040440

**Published:** 2022-03-24

**Authors:** Ahmed M. E. Kotb, Norhan K. Abd El-Aziz, Eman Y. T. Elariny, Reham Yahya, Dalal Hussien M. Alkhalifah, Rania M. Ahmed

**Affiliations:** 1 Department of Botany and Microbiology, Faculty of Science, Zagazig University, Zagazig 44511, Egypt; emanariny@yahoo.com (E.Y.T.E.); raniamuhammad83@gmail.com (R.M.A.); 2 Department of Microbiology, Faculty of Veterinary Medicine, Zagazig University, Sharkia, Zagazig 44511, Egypt; 3 Medical Microbiology, College of Science and Health Professions, King Saud bin Abdulaziz University for Health Sciences, Riyadh 11671, Saudi Arabia; yahyar@ksau-hs.edu.sa; 4 King Abduallah International Medical Research Center, Riyadh 11671, Saudi Arabia; 5 Department of Biology, College of Science, Princess Nourah bint Abdulrahman University, P.O. Box 84428, Riyadh 11671, Saudi Arabia; dhalkalifah@pnu.edu.sa

**Keywords:** urinary tract infection, zinc oxide nanoparticles, *Enterobacterales*, integron, green therapy

## Abstract

Urinary tract infection (UTI) is one of the most common bacterial infections in the world, which is associated with high morbidity and mortality rates. *Enterobacterales* species are considered the most causative agent for UTI, especially uropathogenic *Escherichia coli* (UPEC). Here, we investigated the antibacterial activity of the green fungal metabolite, 6-pentyl α pyrone lactone, alone or in combination with zinc oxide nanoparticles (ZnONPs) against multidrug-resistant *Enterobacterales* recovered from UTI. The results revealed that 57.27% of human urine samples were positive for *Enterobacterales*, where *E. coli* was the most prevalent bacterial pathogen (66.67%). Of note, 98.41% of *Enterobacterales* isolates were multidrug-resistant (MDR) with multiple antimicrobial resistance (MAR) indices ranged from 0.437 to 1. Fifty percent of the examined isolates were positive for the integrase gene; 60% out of them harbored class 2 integron, whereas the other 40% carried class 1 integrons. The broth microdilution assay ensured that the 6-pentyl-α-pyrone lactone had a reasonable antimicrobial effect against the examined isolates (Minimum inhibitory concentration (MIC) values of 16–32 μg/mL). However, ZnONPs showed a strong antimicrobial effect against the investigated isolates with MIC values ranging from 0.015 to 32 μg/mL. Interestingly, the MICs decreased 5–12 fold and 3–11 fold for 6-pentyl-α-pyrone lactone and ZnONPs, respectively, against examined isolates after their combination. This is the first report suggesting the use of 6-pentyl α pyrone lactone and ZnONPs combination as a promising candidate against MDR *Enterobacterales* recovered from UTI.

## 1. Introduction

Urinary tract infection (UTI) is a common worldwide disease. Symptoms are frequently accompanied by difficult urination, burning, and inflammations, which may develop into cystitis, leading to renal failure and further complications. Severity of the disease may increase the hospitalization period, which sustains more financial costs and may be associated with high morbidity and mortality rates [1]. *Enterobacterales* species represent the most infection cause, particularly the uropathogenic *Escherichia coli* (UPEC), which involves more than 80% of all UTIs [2]. Other *Enterobacterales* members such as *Klebsiella*, *Citrobacter* and *Proteus* species are also incriminated in UTI infections.

*Enterobacterales* could acquire antimicrobial resistance properties through various resistance genes, which may be transmitted between bacterial isolates by determinants known as mobile genetic elements (MGE) such as integrons [3]. The integron is an arrangement of gene cassettes. It possesses an integrase gene (*intI*), which encodes a site-specific recombinase, acting as a reservoir for resistance-associated genes, and a specific promoter that is responsible for the expression of any appropriately integrated gene. Integrons were classified into many classes; class I and class II are commonly identified among *Enterobacterales* [4]. These integrons are located on either bacterial plasmids or chromosomes and strongly related to multidrug resistance (MDR) in *Enterobacterales* species [5]. High resistance of *Enterobacterales* to the commonly used antibiotics are considered a severe health and economic problem [6]. Thus, there is an urgent need to develop alternative avenues as herbal compounds or nanomaterial-based approaches for countering antimicrobial resistance in *Enterobacterales* [7].

Green therapy with naturally bioactive compounds is a good way to fight bacterial resistance because of their safety and efficiency. The green fungal metabolite, 6-pentyl-α-pyrone lactone that is produced by *Trichoderma* species showed a strong antifungal activity [8], but their antibacterial activity is still under study. Therefore, we aimed to investigate the activity of 6-pentyl-α-pyrone lactone either alone or in combination with zinc oxide nanoparticles (ZnONPs) against *Enterobacterales* species isolated from human urine samples, which may be a promising alternative to the antimicrobial agents.

## 2. Materials and Methods

### 2.1. Clinical Urine Samples

One hundred and ten human urine samples of both sex [females (*n* = 70) and males (*n* = 40)] were collected from different hospitals and medical laboratories in Zagazig City, Sharkia Governorate, Egypt during the period from January to August 2020. Those samples were categorized into young adult (*n* = 54), middle adult (*n* = 27), old adult (*n* = 13), child (*n* = 9), and early adolescence (*n* = 7). All urine samples were collected from UTI patients; these samples included pus cells, nitrite and many epithelial cells. Samples were placed in sterile urine cups, kept in an icebox packed with ice and directly transferred to the Microbiology laboratory, Faculty of Veterinary Medicine, Zagazig University for bacteriological examination and further analyses. The study was conducted following the Ethics of the World Medical Association (Declaration of Helsinki). Written informed consent was obtained from the patients for participation in this study.

### 2.2. Bacteriological Examination

One mL from each collected sample was added to 9 mL of buffered peptone water (BPW; Conda, Madrid, Spain) for pre-enrichment of human urine samples. A loopful from each pre-enrichment urine sample was cultured onto MacConkey’s agar (HI media, India); then, the growing colonies were sub-cultured on eosin-methylene blue (EMB; HI media, India) agar media [9]. Various biochemical tests as Simmons’ citrate, urease and indole, as well as the characteristic reactions on triple sugar iron (TSI; Oxoid, UK) agar media were examined according to Finegold et al. [10] for further differentiation of *Enterobacterales* members. Polymerase chain reaction (PCR)-based confirmation of *Enterobacterales* was applied using oligonucleotide primers listed in Table S1 [11,12,13,14].

### 2.3. Antimicrobial Susceptibility Testing

Antimicrobial susceptibility testing of *Enterobacterales* isolates was done, adopting the standardized disc diffusion method [15]. Sixteen widely used antimicrobial agents of nine antimicrobial classes were tested (Bioanalyse, Ankara, Turkey). They included cefepime (30 μg), cefuroxime (30 μg), cefotaxime (30 μg), imipenem (10 μg), meropenem (10 μg), amoxicillin/clavulanic acid (20/10 μg), piperacillin/tazobactam (100/10 μg), ampicillin/sulbactam (10/10 μg), trimethoprim/sulphamethoxazole (1.25/23.75 μg), doxycycline (30 μg), erythromycin (15 μg), levofloxacin (5 μg), ciprofloxacin (5 μg), gentamicin (10 μg), amikacin (30 μg), and nitrofurantoin (300 μg). The inhibition zone diameters were interpreted according to the Clinical and Laboratory Standards Institute and European Committee on Antimicrobial Susceptibility Testing [16,17] guidelines. Bacterial isolates showing resistance to ≥three antimicrobial classes were considered MDR. Multiple antibiotic resistance (MAR) index was determined for each isolate by calculating the number of antimicrobials showed resistance/total number of tested antimicrobial agents, while the MAR index for each antimicrobial = total number of recorded resistance/(total number of tested antimicrobials × total number of isolates) [18].

### 2.4. Plasmid Extraction and Detection of the Integrase Gene

Plasmid extraction of MDR *Enterobacterales* isolates was done using the QIAprep Spin Miniprep Kits according to the manufacturer’s instructions (Qiagen, Gmbh, Germany). Conventional PCR was applied to hybridize the conserved regions of the integrase encoded genes, *intI1* and *intI2*, using hep35 and hep36 [19] oligonucleotide primers presented in Table S1. PCR amplifications were performed with a total volume of 25 μL of the following reaction mixture: 12.5 μL DreamTaq Green PCR Master Mix (2X) (Thermo Fisher Scientific, Waltham, MA, USA), 1 μL of each primer (20 pmole), 2 μL template DNA and 8.5 μL water nuclease-free.

### 2.5. Restriction Fragment Length Polymorphism (RFLP) for Integrons Categorization

Using *Rsa*I restriction Enzyme 11, the PCR products were digested, then class 1 integron cassette structures were amplified using hep58 and hep59 primer segments, while class 2 integrons were amplified using hep74 and hep51 primer regions (Table S1). PCR amplifications were performed using a PTC-100^TM^ programmable thermal cycler (MJ Research Inc., Waltham, MA, USA) as described elsewhere [19]. A positive control (an integrase positive *E. coli* isolate) and a negative control (Master Mix without DNA) were included. PCR amplicons were separated by electrophoresis on 1.5% agarose gel (Sigma-Aldrich, St. Louis, MO, USA) stained with 0.5 μg/mL ethidium bromide (Sigma-Aldrich, USA). A gene ruler 100 bp DNA ladder (Thermofisher Scientific, Waltham, MA, USA) was used to measure the fragment sizes of class 1 (491 bp) and class 2 (334 bp and 157 bp fragments) integrons.

### 2.6. Preparation of 6-Pentyl-α-Pyrone Lactone and Zinc Oxide Nanoparticles

A stock solution of 100% commercially available 6-pentyl-α-pyrone lactone (Sigma Aldrich, Germany) was prepared by dissolving in methanol (98%; ALPHA Chemika, Mumbai, India) according to Ismaiel et al. [8]. Synthesized ZnONPs of spherical shape, with an average size of 54.53 nm and a specific surface area of 20.28 m^2^ g^−1^ [20] were purchased from Naqaa Co. (Cairo, Egypt). The ZnONPs stock solution was prepared as 1 μg/mL by dissolving in a desired volume of sterile distilled water.

### 2.7. Antimicrobial Activities of 6-Pentyl-α-Pyrone Lactone, Zinc Oxide Nanoparticles and Their Combination

The activities of 6-Pentyl-α-pyrone lactone and ZnONPs against MDR *Enterobacterales* isolates were screened by the agar well diffusion method, as described previously [21]. Minimum inhibitory concentrations (MICs) and minimum bactericidal concentrations (MBCs) of 6-pentyl-α-pyrone lactone and ZnONPs were determined by the broth microdilution technique according to Rankin [22]. The interaction activities of antimicrobial combination were assessed by the checkerboard method [23] using Muller–Hinton broth (Oxoid, Hampshire, UK) and a bacterial density of 5 × 10^5^ CFU/mL. Fractional inhibitory concentrations (FIC) of the antimicrobial combination were calculated as mentioned by Hsieh et al. [24]. The combination is considered synergistic when the FIC index (ΣFIC) is ≤0.5, indifferent when the ΣFIC is >0.5 to <2, and antagonistic when the ΣFIC is ≥2. The MIC 50 and MIC 90 were calculated using an orderly array method [25].

### 2.8. Statistical Analysis

The data presented in the study were analyzed in Microsoft Excel software (Microsoft Corporation, Redmond, WA, USA). Sample size was detected according to Thompson equation at CI = 95%, Z = 1.96, α = 0.05, D = 0.05 and *p* = 0.50 [26]. Binary logistic regression analysis (PROC LOGISTIC; SAS Institute Inc., Cary, NC, USA) was run setting the level of significance at α = 0.05 to examine the effects of the potential risk factors including age and sex on *Enterobacterales* occurrence [27]. Significant differences in antimicrobial susceptibilities of *Enterobacterales* isolates, as well as the differences among explanatory variables, were tested via Fisher’s Exact Test. The differences between MIC means of each examined antimicrobial agent and their combination were separated by Tukey’s studentized range (HSD) test. Statistical significance was set at *p*-value less than 0.05.

## 3. Results

### 3.1. Occurrence of Enterobacterales in Clinical Urine Samples

As presented in Table 1, sixty-three *Enterobacterales* isolates were recovered from 110 human urine samples (57.27%), which were more frequent in females (*n* = 52; 74.29%) than in males (*n* = 11; 27.50%) (*p* < 0.05). Young adults represented the most common cases (75.47%), followed by old adults (53.85%), and middle adults (42.86%), whereas young ages, e.g., childhood and early adolescence, represented the lowest infectious cases (33.33% and 14.29%, respectively). *Enterobacterales* isolates were classified into four species; *E. coli*, which was the most prevalent bacterial pathogen (66.67%), followed by *Klebsiella* (28.57%), *Citobacter* (3.17%) and *Proteus* (1.58%) species. Higher frequencies of *E. coli* and *Klebsiella* species were observed in the young adulthood period and females. The probability of *Enterobacterales* occurrence decreased by 85% (0.153), 74% (0.266), 67% (0.33), and 5% (0.952) during the periods of older adulthood, middle adulthood, early adolescence, and young adulthood, respectively, compared to the childhood period. With regard to sex, males had 80% (0.200) lower odds of *Enterobacterales* occurrence than females.

### 3.2. Antimicrobial Susceptibility Results

The antimicrobial susceptibilities of *Enterobacterales* isolates (*n* = 63) against 16 broadly used antimicrobial agents of various antimicrobial classes (*n* = 9) are depicted in Table 2. The lowest resistance percentage was reported for meropenem (38.09%), nitrofurantoin (41.26%) and imipenem (46.03%). Nevertheless, high resistance level was observed with cefotaxime (100%), followed by cefepime (96.82%), cefuroxime (95.23%), erythromycin (92.05%), ciprofloxacin (84.12%), piperacillin/tazobactam (82.53%), amoxicillin-clavulanic acid (79.36%), and levofloxacin (77.77%). Sixty-two (98.41%) *Enterobacterales* isolates were categorized as MDR; they exhibited resistant to more than three antimicrobial classes, and their MAR indices were greater than 0.4 (0.437–1). Statistical analysis revealed significant differences (*p* = 0.001) in antimicrobial susceptibilities of *Enterobacterales* isolates recovered from clinical urine samples for all antimicrobial agents except for meropenem (*p* = 0.404) and nitrofurantoin (*p* = 0.228). 

### 3.3. Existence of the Integrase Gene among MDR Enterobacterales Isolates

**Figure 1:**
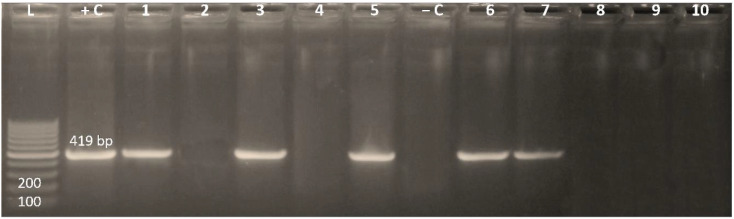
Agarose gel electrophoresis of the integrase gene among *Enterobacterales* isolates. Lane L: 100-bp ladder; +C: positive control; −C: negative control; lanes 1, 3, 5, 6 and 7: positive integrase targeted at 491 bp.

Multidrug-resistant *Enterobacterales* isolates with high MAR indices (0.687–1; *n* = 10) were screened for the presence of the integrase gene (*intI*) located on plasmid by conventional PCR. Fifty percent of the examined isolates were positive for the integrase gene (Figure 1). Of note, 20% of the positive isolates were *E. coli*, while the higher prevalence of *intI* gene was recorded for *Klebsiella* species (80%). 

### 3.4. Detection of Class 1 and Class 2 Integrons by PCR-RFLP

**Figure 2:**
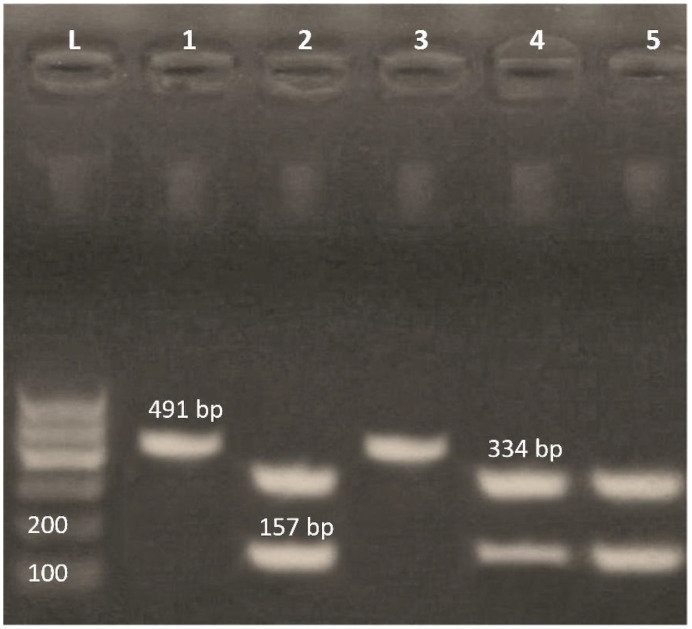
PCR-RFLP assay for differentiation of class 1 and class 2 integrons using *Rsa*I restriction enzyme. Lane L: 100-bp ladder; Lanes 2, 4 and 5 (334 bp and 157 bp) represent class II integrons; Lanes 1 and 3 (491 bp) represent class I integrons.

Positive integrase isolates (*n* = 5) were screened for the presence of class 1 and class 2 integrons by PCR-RFLP. The results revealed that 60% of isolates harbored class 2 (fragments’ sizes = 157 and 334 bp), while 40% of the isolates carried class 1 integrons (product size = 491 bp) (Figure 2). 

### 3.5. Antimicrobial Activities of 6-Pentyl-α-Pyrone Lactone and Zinc Oxide Nanoparticles against MDR Enterobacterales Isolates

Six-pentyl-α-pyrone lactone fungal metabolite and ZnONPs were tested against MDR *Enterobacterales* of high MAR indices, including those harbored integrons (Table 3). The results showed that all isolates were resistant to 6-pentyl-α-pyrone lactone using agar well diffusion method, while ZnONPs was effective against all tested isolates (inhibition zone diameters are ≥15 mm). The broth microdilution assay ensured that the 6-pentyl-α-pyrone had a weak antimicrobial effect against the examined isolates with MIC values of 16–32 μg/mL. However, ZnONPs showed a strong antimicrobial effect against the investigated isolates with MIC values ranging from 0.015 to 32 μg/mL. Moreover, ZnONPs were reported to have a strong bactericidal activity against 60% of examined isolates with MIC similar to MBC for *E.coli* (16 μg/mL) and *Klebsiella* (8 μg/mL) species. Checkerboard assay was applied for determination of the antimicrobial activity of 6-pentyl-α-pyrone lactone and ZnONPs combinations against the MDR *Enterobacterales* isolated from human urine samples. As mentioned in Table 3, the ΣFIC revealed synergism activity for 90% of the examined isolates, while the latest 10% displayed indifference activity. Of note, the MICs decreased 5–12 fold and 3–11 fold for 6-pentyl-α-pyrone lactone and ZnONPs, respectively against the examined isolates after their combination. MIC 50 and MIC 90 of 6-pentyl-α-pyrone lactone, ZnONPs and their combination against analyzed isolates are shown in Table 4.

## 4. Discussion

Multidrug resistance in UTI patients is considered a common healthcare problem [28]. Such resistance in cases of *Enterobacterales* infection may increase the mortality rate due to limited medication, which developed into a long residence in hospitals leading to financial load [29,30].

In this study, 57.27% of *Enterobacterales* isolates were isolated from UTI in patients, with a higher percentage in females (74.29%) than in males (27.50%), which was consistent with that recorded previously [31], and contrary to that reported by Elshamy et al. [32], who revealed 43.4% *Enterobacterales* isolates in females and 56.6% in males. This variation may be attributed to the UTI risk factors or geographical distribution. In addition, the young adults showed the most infection cases here, that was in conformity with the previously reported results in Upper Egypt [33], which may relate to the UTI incidence in this age or age variations. In the current study, *E. coli* represents the most prevalent pathogen (66.67%), followed by *Klebsiella* species (28.57%) as reported previously in Egypt (38.69 and 21.35%, respectively) [34], and in Turkey (71.7 and 10.7%, respectively) [35]. 

The development of bacterial resistance to various antimicrobials has become a grave threat, as there are fewer effective antimicrobial agents helpful for treating these organisms. Herein, the antimicrobial susceptibility testing revealed that cefotaxime showed the highest resistance rate against *Enterobacterales* (100%), followed by cefepime, cefuroxime, erythromycin, ciprofloxacin and piperacillin/tazobactam (≥80% of isolates), which is in conformity with recently published researches [36,37]. High sensitivity level was observed for meropenem, nitrofurantoin, and imipenem, which was similar to those reported earlier in Egypt [36,38] and Ethiopia [39]. Also, MAR indices were more than 0.4 in this study for all resistant isolates, which agreed with what was mentioned previously in Egypt [40,41,42] and in Iraq [43] from different clinical samples where urine was included. Integrons are considered a fundamental cause of multiple antimicrobial resistance gene cassettes transmission in Gram-negative bacteria causing MDR phenotype [44]. In the current study, integron genes located on plasmids were presented in 50% of examined isolates, which potentially reflect the transmission of resistance genes among isolates. Abdel-Rhman and coauthors [45] documented nearly similar results (44%) in Mansoura, Egypt. Of interest, class 2 integron was more frequent than class 1, which is contrary to a recent study [46] in which the class 1 and class 2 percentages were 50% and 2.4%, respectively. Another study [47] in Nigeria showed that only class 1 integron was detected.

Six-pentyl-α-pyrone lactone is a fungal metabolite purified from *Trichoderma* species, which has an antimicrobial effect. In our study, 6-pentyl-α-pyrone lactone exhibited lower antibacterial activity in both agar well diffusion and broth microdilution assay against *Enterobacterales* isolates. The same results were previously reported in Egypt [8] against a standard *E. coli* strain (ATCC 11229) and a *klebsiella* isolate sourced from urine samples. ZnONPs have emerged a promising prospective in biomedicine, particularly in anticancer and antibacterial fields. Previous studies proved that ZnONPs have become one of the most prevalent metal oxide nanoparticles in biological applications due to their brilliant biocompatibility economic, and low toxicity [48]. Herein, ZnONPs provided a strong antimicrobial effect against tested isolates of *Enterobacterales* (*E. coli* and *Klebsiella*). Similar results were documented previously in Egypt [7] against *E. coli* and *Klebsiella* strains isolated from UTI patients.

In this study, six-pentyl-α-pyrone lactone and ZnONPs combination success to develop a greater activity (synergism) than each one alone in 90% of tested isolates. The small size of ZnONPs may help the combined 6-pentyl-α-pyrone lactone to enter the bacterial cell and express its antimicrobial effect. In addition to the action of ZnONPs against bacterial strains, their combination with the fungal metabolite could decrease the MICs to 11–12 fold, suggesting a new promising candidate for treating MDR bacteria incriminated in UTI.

## 5. Conclusions

Multidrug resistance among *Enterobacterales* species causing UTI is a severe problem that is developed in our country and needs more attention. Six-pentyl-α-pyrone lactone and its synergistic effect with ZnONPs against MDR *Enterobacterales* species may be promising agents to overcome increasing resistance as a first report. The knowledge gained from this study is the in vitro preliminary validation of the fungal metabolite and nanoparticles for the mitigation of bacterial resistance. However, no method supports the clinical use of these compounds in UTI without in vivo studies.

## Figures and Tables

**Table 1:** Occurrence of *Enterobacterales* in human urine samples.

Enterobacterales Species	Age					Sex	
	C (n = 9)	EA (n = 7)	YA (n = 53)	MA (n = 28)	OA (n = 13)	F (n = 70)	M (n = 40)
*E. coli*	2 (22.22)	1 (14.29)	23 (43.39) *	11 (39.28)	5 (38.46)	33 (47.14) *	9 (30)
*Klebsiella*	1 (11.11)	-	16 (30.18) *	-	1 (7.69)	17 (24.28) *	1 (3.33)
*Citrobacter*	-	-	1 (1.88)	1 (3.57)	-	2 (2.85) NE	-
*Proteus*	-	-	-	-	1 (7.69)	-	1 (3.33) NE
Total	3 (33.33) (Ref.)	1 (14.29) (0.33 ¶) *	40 (75.47) (0.952 ¶) ^ns^	12 (42.86) (0.266 ¶) *	7 (53.85) (0.153 ¶) *	52 (74.29) (Ref.)	11 (27.50) (0.200 ¶) *

C, Childhood (0–11 years); EA, Early Adolescence (12–18 years); YA, Young Adulthood (19–44 years); MA, Middle Adulthood (45–64 years); OA, Older Adulthood (65 years and older); F, female, M, male; (-), not detected; ^ns^, non-significant; NE, statistical value none estimated; Ref., reference. Data are represented by frequencies (%). * Significant at *p*-value < 0.05; ¶ represented the odds ratio.

**Table 2:** Antimicrobial susceptibilities of *Enterobacterales* isolates (*n* = 63) recovered from clinical urine samples.

Antimicrobial Agent	Susceptibility *			MAR Index	*p*-Value
	Sensitive	Intermediate	Resistant		
Amoxicillin clavulanic acid (AMC)	7 (11.11)	6 (9.52)	50 (79.36)	0.050	0.001
Ampicillin sulbactam (SAM)	6 (9.52)	11 (17.46)	46 (73.01)	0.045	0.001
Piperacillin tazobactam (TPZ)	7 (11.11)	4 (6.34)	52 (82.53)	0.051	0.001
Amikacin (AK)	17 (26.98)	4 (6.34)	42 (66.67)	0.042	0.001
Gentamycin (CN)	12 (19.04)	6 (9.52)	45 (71.42)	0.044	0.001
Imipenem (IMP)	25 (39.68)	9 (14.28)	29 (46.03)	0.028	0.004
Meropenem (MEM)	23 (36.50)	16 (25.39)	24 (38.09)	0.023	0.404
Doxycycline (DO)	27 (42.85)	3 (4.76)	33 (52.38)	0.033	0.001
Ciprofloxacin (CIP)	7 (11.11)	3 (4.76)	53 (84.12)	0.052	0.001
Levofloxacin (LEV)	10 (15.87)	4 (6.34)	49 (77.77)	0.047	0.001
Trimethoprime + sulfamethaxazole (SXT)	11 (17.46)	6 (9.52)	46 (73.01)	0.047	0.001
Nitrofurantoin (F)	22 (34.92)	15 (23.80)	26 (41.26)	0.027	0.228
Cefuroxime (CXM)	1 (52.17)	2 (00.00)	60 (47.82)	0.059	0.001
Cefepime (FEB)	0 (00.00)	2 (3.17)	61 (96.82)	0.060	0.001
Cefotaxime (CTX)	0 (00.00)	0 (00.00)	63 (100.00)	0.062	NE
Erythromycin (E)	4 (6.34)	1 (1.58)	58 (92.06)	0.057	0.001

Antimicrobial sensitivity cut-off values were determined following CLSI 2020 and EUCAST, 2021. MAR, multiple antibiotic resistance; NE, not estimated. * Data are presented by No. (%). *p*-values < 0.05 are statistically significant.

**Table 3:** MIC results of 6-pentyl-α-pyrone fungal metabolite, zinc oxide nanoparticles, and their combinations against MDR *Enterobacterales* isolates.

Isolate No.	Antimicrobial Resistant Pattern	Bacterial Species	MIC (μg/mL)				Interactive Category
			Fungal Extract	ZnONPs	Fungal Extract/ZnONPs	ΣFIC	
1	SAM, TPZ, AK, CN, IPM, DO, CIP, LEV, SXT, CXM, FEP, CTX, E	*E. coli*	32	32	½	0.0937	Synergism
2	AMC, SAM, TPZ, AK, CN, IPM, MEM, DO, CIP, LEV, SXT, CXM, FEP, CTX, E	*E. coli*	32	0.015	0.0075/0.015	0.5004	Synergism
3	AMC, SAM, TPZ, AK, CN, IPM, MEM, DO, CIP, LEV, SXT, F, CXM, FEP, CTX, E	*Klebsiella*	16	0.015	0.0075/0.015	0.5009	Synergism
4	AMC, SAM, TPZ, CN, DO, CIP, LEV, SXT, F, CXM, FEP, CTX, E	*Klebsiella*	32	0.062	0.0075/0.015	0.1214	Synergism
5	AMC, SAM, CN, IPM, DO, CIP, LEV, SXT, CXM, FEP, CTX, E	*Klebsiella*	32	1	0.0075/0.015	0.0079	Synergism
6	AMC, SAM, TPZ, AK, CN, IPM, MEM, DO, CIP, LEV, SXT, F, CXM, FEP, CTX, E	*Klebsiella*	16	8	0.031/0.062	0.0077	Synergism
7	AMC, SAM, TPZ, AK, CN, IPM, MEM, DO, CIP, LEV, SXT, F, CXM, FEP, CTX, E	*Klebsiella*	32	1	0.0075/0.015	0.0079	Synergism
8	AMC, SAM, TPZ, AK, CN, IPM, MEM, CIP, LEV, SXT, F, CXM, FEP, CTX, E	*E. coli*	32	16	0.0075/0.015	0.00093	Synergism
9	AMC, SAM, TPZ, AK, CN, IPM, MEM, DO, CIP, LEV, SXT, F, CXM, FEP, CTX, E	*E. coli*	32	0.015	0.015/0.031	1.0009	Indifference
10	AMC, SAM, TPZ, AK, CN, IPM, DO, CIP, LEV, SXT, CXM, FEP, CTX, E	*Klebsiella*	16	32	0.0075/0.015	0.0032	Synergism
Means ± SE			27.2 ± 2.44	9.01 ± 4.16 *	0.109 ± 0.09/0.219 ± 0.197 *^,‡^		

MIC, minimum inhibitory concentration; ZnONPs, zinc oxide nanoparticles; ΣFIC, fractional inhibitory concentrations index. The antimicrobial agents are considered to have synergistic activity if the ΣFIC value is less than or equal 0.5. The effect is considered to be additive, if the ΣFIC value is more than 0.5 but less than or equal to 1.0 (ΣFIC > 0.5 but ≤1). The effects are considered indifferent when the value lies between 1.0 and 4.0. The agents are considered to possess antagonistic activity if the value of ΣFIC is ≥4.0: SE; standard error; * differ significantly with fungal extract (*p* < 0.05): **^‡^** differ significantly with ZnONPs (*p* < 0.05).

**Table 4:** MIC 50 and MIC 90 of the fungal metabolite, zinc oxide nanoparticles and their combination.

MIC	Antibacterial Agents (μg/mL)		
	Fungal Metabolite	ZnONPs	Combinations of ZnONPs and Fungal Metabolite
MIC range	16–64	0.015–32	0.0075/0.015-½
MIC 50 ^a^	32	1	0.0075/0.015
MIC 90 ^b^	16	0.015	0.0075/0.015

MIC, minimum inhibitory concentration; ^a^ MIC 50, the MIC at which 50% of the bacterial cells are inhibited; ^b^ MIC 90, the MIC at which 90% of the bacterial cells are inhibited; ZnONPs, zinc oxide nanoparticles.

## Data Availability

Primary sequence data analyzed in this study and related information are available in Supplementary Materials.

## Supplementary Materials

The following supporting information can be downloaded at: https://www.mdpi.com/article/10.3390/antibiotics11040440/s1. Table S1: Oligonucleotide primer sequences used in this study.

## References

[1] Kalra O.P., Raizada A. (2009). Approach to a patient with urosepsis. J. Glob. Infec. Dis..

[2] Walters M.S., Lane M.C., Vigil P.D., Smith S.N., Walk S.T., Mobley H.L. (2012). Kinetics of uropathogenic *Escherichia coli* metapopulation movement during urinary tract infection. MBio.

[3] Partridge S.R., Kwong S.M., Firth N., Jensen S.O. (2018). Mobile genetic elements associated with antimicrobial resistance. Clin. Microbiol. Rev..

[4] Carattoli A. (2001). Importance of integrons in the diffusion of resistance. Vet. Res..

[5] Leverstein-van Hall M.A., Blok H.E.M., Donders A.R.T., Paauw A., Fluit A.C., Verhoef J. (2003). Multidrug resistance among *Enterobacteriaceae* is strongly associated with the presence of integrons and is independent of species or isolate origin. J. Infec. Dis..

[6] Kaminska P.S., Yernazarova A., Murawska E., Swiecicki J., Fiedoruk K., Bideshi D.K., Swiecicka I. (2014). Comparative analysis of quantitative reverse transcription real-time PCR and commercial enzyme imunoassays for detection of enterotoxigenic *Bacillus thuringiensis* isolates. FEMS Microbiol. Lett..

[7] El-Rab S.M.G., Abo-Amer A.E., Asiri A.M. (2020). Biogenic synthesis of ZnO nanoparticles and its potential use as antimicrobial agent against multidrug-resistant pathogens. Curr. Microbiol..

[8] Ismaiel A.A., Ali D.M. (2017). Antimicrobial properties of 6-pentyl-α-pyrone produced by endophytic strains of *Trichoderma koningii* and its effect on aflatoxin B1 production. Biologia.

[9] Hall H.E., Brown D.F., Lewis K.H. (1967). Examination of market foods for coliform organisms. Appl. Microbiol..

[10] Finegold S.M., Martin W.J., Scott E.G. (1978). Bailey and Scott’s Diagnostic Microbiology.

[11] Heijnen L., Medema G. (2006). Quantitative Detection of E. Coli, E. Coli O157 and Other Shiga Toxin Producing E. Coli in Water Samples Using a Culture Method Combined with Real-Time PCR. J. Water Health.

[12] Brisse S., Verhoef J. (2001). Phylogenetic diversity of *Klebsiella pneumoniae* and *Klebsiella oxytoca* clinical isolates revealed by randomly amplified polymorphic DNA, *gyrA* and *parC* genes sequencing and automated ribotyping. Int. J. Syst. Evol. Microbiol..

[13] Anbazhagan D., Kathirvalu G.G., Mansor M., Yan G.O.S., Yusof Y.M., Sekaran S.D. (2010). Multiplex Polymerase Chain Reaction (PCR) Assays for the Detection of *Enterobacteriaceae* in Clinical Samples. Afr. J. Microbiol. Res..

[14] Bi S., Tang S., Wu X., Chen S. (2013). Quantitative detection of Proteus species by real-time polymerase chain reaction using SYBR Green. Ann. Microbiol..

[15] Bauer A.W. (1966). Antibiotic susceptibility testing by a standardized single disc method. Am. J. Clin. Pathol..

[16] Clinical and Laboratory Standards Institute (2020). Performance Standards for Antimicrobial Susceptibility Testing.

[17] EUCAST: The European Committee on Antimicrobial Susceptibility Testing (2021). Breakpoint Tables for Interpretation of MICs and Zone Diameters Version 11.0. http://www.eucast.org.

[18] Tambekar D.H., Dhanorkar D.V., Gulhane S.R., Khandelwal V.K., Dudhane M.N. (2006). Antibacterial susceptibility of some urinary tract pathogens to commonly used antibiotics. Afr. J. Biotechnol..

[19] White P.A., McIver C.J., Deng Y.M., Rawlinson W.D. (2000). Characterization of two new gene cassettes, *aadA5* and *dfrA17*. FEMS Microbiol. Lett..

[20] Suntako R. (2015). Effect of zinc oxide nanoparticles synthesized by a precipitation method on mechanical and morphological properties of the CR foam. Bull. Mat. Sci..

[21] Valgas C., Machado de Souza S., Smânia E.F.A., Smânia A.J.R. (2007). Screening methods to determine antibacterial activity of natural products. Braz. J. Microbiol..

[22] Rankin I.D. (2005). MIC testing. Manual of antimicrobial susceptibility testing. American Society for Microbiology.

[23] Yadav M.K., Park S.W., Chae S.W., Song J.J., Kim H.C. (2013). Antimicrobial activities of *Eugenia caryophyllata* extract and its major chemical constituent eugenol against *Streptococcus pneumonia*. Acta Pathol. Microbiol. Immunol. Scand..

[24] Hsieh M.H., Chen M.Y., Victor L.Y., Chow J.W. (1993). Synergy assessed by checkerboard a critical analysis. Diagn. Microbiol. Infect.Dis..

[25] Hamilton-Miller J.M.T. (1991). Calculating MIC 50. J. Antimicrob. Chemother..

[26] Thompson S. (2012). Sampling.

[27] SAS Institute Inc. (2012). SAS/STAT Statistics user’s guide. Statistical Analytical System.

[28] Podschun R., Ullmann U. (1998). *Klebsiella* spp. as nosocomial pathogens: Epidemiology, taxonomy, typing methods, and pathogenicity factors. Clin. Microbiol. Rev..

[29] Navon-Venezia S., Kondratyeva K., Carattoli A. (2017). *Klebsiella pneumoniae*: A major worldwide source and shuttle for antibiotic resistance. FEMS Microbiol. Rev..

[30] Abdelwahab R., Yasir M., Godfrey R.E., Christie G.S., Element S.J., Saville F., Browning D.F. (2021). Antimicrobial resistance and gene regulation in Enteroaggregative *Escherichia coli* from Egyptian children with diarrhoea: Similarities and differences. Virulence.

[31] Kishk R. (2021). Bacterial Pattern of Community acquired Urinary Tract Infections: A Challenge for Antimicrobial Resistance. Egypt. J. Med. Microbiol..

[32] Elshamy A.A., Saleh S.E., Alshahrani M.Y., Aboshanab K.M., Aboulwafa M.M., Hassouna N.A. (2021). OXA-48 Carbapenemase-Encoding Transferable Plasmids of *Klebsiella pneumoniae* Recovered from Egyptian Patients Suffering from Complicated Urinary Tract Infections. Biology.

[33] Kotb D.N., Mahmoud M.S., Mahdi W.K., Khairy R.M. (2019). Prevalence and Antimicrobial Resistance of Urinary Tract Infections in Upper Egypt. MJMR.

[34] Desouky D.E., Gabr H.M., El-Helbawy M., Hathout H.M. (2020). Urinary Tract Infection: Prevalence, Risk Factors, Bacterial Etiologies and Antimicrobial Resistance Profile among Egyptian Diabetic Patients: Urinary Tract Infection: Prevalence, Risk Factors, Bacterial Etiologies and Antimicrobial Resistance Profile among Egyptians. Eur. J. Med. Health Sci..

[35] Öztürk R., Tazegul G. (2021). Bacteria Causing Community-Acquired Urinary Tract Infections and Their Antibiotic Susceptibility Patterns in Outpatients Attending at a State Hospital in Turkey. Cureus.

[36] Said A., El-Gamal M.S., Abu-Elghait M., Salem S.S. (2021). Isolation, Identification and Antibiotic Susceptibility Pattern of Urinary Tract Infection Bacterial Isolates. Lett. Appl. NanoBioSci..

[37] Tartor Y.H., Abd El-Aziz N.K., Gharieb R.M.A., El Damaty H.M., Enany S., Soliman E.A., Abdellatif S.S., Attia A.S.A., Bahnass M.M., El-Shazly Y.A. (2021). Whole-Genome Sequencing of Gram-Negative Bacteria Isolated From Bovine Mastitis and Raw Milk: The First Emergence of Colistin *mcr*-*10* and Fosfomycin *fosA5* Resistance Genes in *Klebsiella pneumoniae* in Middle East. Front. Microbiol..

[38] Shash R.Y., Elshimy A.A., Soliman M.Y., Mosharafa A.A. (2019). Molecular Characterization of Extended-Spectrum β-Lactamase *Enterobacteriaceae* Isolated from Egyptian Patients with Community- and Hospital-Acquired Urinary Tract Infection. Am. J. Trop. Med. Hyg..

[39] Legese M.H., Weldearegay G.M., Asrat D. (2017). Extended-spectrum beta-lactamase-and carbapenemase-producing *Enterobacteriaceae* among Ethiopian children. Infec. Drug Resis..

[40] Elmowalid G.A., Ahmad A.A.M., Hassan M.N., Abd El-Aziz N.K., Abdelwahab A.M., Elwan S.I. (2018). Molecular detection of new SHV β-lactamase variants in clinical *Escherichia coli* and *Klebsiella pneumoniae* isolates from Egypt. Comp. Immunol. Microbiol. Infect. Dis..

[41] Elmonir W., El-Aziz A., Norhan K., Tartor Y.H., Moustafa S.M., Abo Remela E.M., Eissa R., Saad H.A., Abdel Tawab H. (2021). Emergence of Colistin and Carbapenem Resistance in Extended-Spectrum β-Lactamase Producing *Klebsiella pneumoniae* Isolated from Chickens and Humans in Egypt. Biology.

[42] Tartor Y.H., Gharieb R.M.A., Abd El-Aziz N.K., El Damaty H.M., Enany S., Khalifa E., Attia A.S.A., Abdellatif S.S., Ramadan H. (2021). Virulence determinants and plasmid-mediated colistin resistance mcr genes in Gram-negative bacteria isolated from bovine milk. Front. Cell. Infect. Microbiol..

[43] Lazm A.M., Al-Allak M.H., Al-Asskar J.A., Al-Dahmoshi H.O., Al-Khafaji N.S. (2019). Antibiotics resistance patterns among *Enterobacteriaceae* isolated from different clinical samples. Drug Invent. Today.

[44] Abd El-Aziz N.K., Ammar A.M., Hamdy M.M., Gobouri A.A., Azab E., Sewid A.H. (2020). First Report of *aacC5-aadA7Δ4* Gene Cassette Array and Phage Tail Tape Measure Protein on Class 1 Integrons of *Campylobacter* Species Isolated from Animal and Human Sources in Egypt. Animals.

[45] Abdel-Rhman S.H., Elbargisy R.M., Rizk D.E. (2021). Characterization of Integrons and Quinolone Resistance in Clinical *Escherichia coli* Isolates in Mansoura City, Egypt. Int. J. Microbiol..

[46] Khalifa H.O., Soliman A.M., Ahmed A.M., Shimamoto T., Nariya H., Matsumoto T., Shimamoto T. (2019). High prevalence of antimicrobial resistance in Gram-negative bacteria isolated from clinical settings in Egypt: Recalling for judicious use of conventional antimicrobials in developing nations. Microb. Drug Resis..

[47] Barns J.N., Ezeamagu C.O., Nkemjika M.E., Akindele T.S. (2021). Prevalence of integrons in *Enterobacteriaceae* obtained from clinical samples. J. Microbiol. Antimicrob..

[48] Jiang J., Pi J and Cai J. (2018). The Advancing of Zinc Oxide Nanoparticles for Biomedical Applications. Bioinorg. Chem. Appl..

