# Supplementary material for: Synergistic Antibacterial Potential of 6-Pentyl-α-pyrone Lactone and Zinc Oxide Nanoparticles against Multidrug-Resistant Enterobacterales Isolated from Urinary Tract Infections in Humans
Source: Antibiotics (Basel). 2022 Mar 24;11(4):440. doi: 10.3390/antibiotics11040440 (PMC9027854; doi:10.3390/antibiotics11040440)
Supplement: Supplementary file 1 [file antibiotics-11-00440-s001.zip › antibiotics-1627518-supplementary.pdf]

Table S1: Oligonucleotide primer sequences used in this study

Primer use and target gene	Nucleotide sequence (5′→3′)	Amplicon size (bp)	Annealing temperature (°C)	Reference
**Bacterial identification:**				
E. coli uidA	F: TATGGAATTTCGCCGATTTT<br>R: TGTTTGCCTCCCTGCTGCGG	166	55	[11]
Klebsiella species *gyrA*	F: CGCGTACTATACGCCATGAACGTA<br>R: ACCGTTGATCACTTCGGTCAGG	441	50	[12]
Citrobacter species*16S rRNA*	F: GCTCAACCTGGGAACTGCATCCGA<br>R: AGTTCCGGCCTAACCGCTGGCAA	529	58	[13]
Proteus species *atpD*	F: GTATCATGAACGTTCTGGGTAC<br>R:TGAAGTGATACGCTCTTGCAG	595	58	[14]
Integrase gene (*intI*)	hep35: TGCGGGTYAARGATBTKGATTT<br>hep36: CAR CACATGCGTRTARAT	491	55	[19]
Class 1 integron	hep58: TCATGGCTTGTTATGACTGT<br>hep59: GTAGGGCTTATTATGCACGC	491	55	[19]
Class 2 integron	hep74: CGGGATCCCGGACGGCATGCACGATTTGTA<br>hep51: GATGCCATCGCAAGTACGAG	157; 334	55	[19]

F, forward; R, reverse; bp, base pair
B = C or G or T, K = G or T, R = A or G and Y = C or T.
